# Walnut Polyphenol Extract Protects against Fenitrothion-Induced Immunotoxicity in Murine Splenic Lymphocytes

**DOI:** 10.3390/nu10121838

**Published:** 2018-11-30

**Authors:** Hong Liu, Yifang Wan, Yuxin Wang, Yue Zhao, Yue Zhang, Ao Zhang, Qiang Weng, Meiyu Xu

**Affiliations:** 1 Collage of Biological Science and Technology, Beijing Forestry University, Beijing 100083, China; liuhong@bjfu.edu.cn (H.L.); wanyifang666@bjfu.edu.cn (Y.W.); wangyuxin@bjfu.edu.cn (Y.W.); zhaoyue7180171@bjfu.edu.cn (Y.Z.); zy15810252987@bjfu.edu.cn (Y.Z.); zhangaoo@bjfu.edu.cn (A.Z.); qiangweng@bjfu.edu.cn (Q.W.); 2 Beijing Key Laboratory of Forest Food Processing and Safety, Beijing Forestry University, Beijing 100083, China

**Keywords:** Fenitrothion, walnut polyphenol extract, immunotoxicity, splenic T-lymphocytes, oxidative stress

## Abstract

Fenitrothion (FNT), an organophosphate pesticide, exerts an immunotoxic effect on splenocytes. Dietary polyphenol compounds exert antioxidant, anticancer and antihypertensive effects. In this study, we investigated the effect of walnut polyphenol extract (WPE) on FNT-induced immunotoxicity in splenic lymphocytes in vitro. Treatment with WPE significantly increased the proliferation of FNT-exposed splenocytes, as evidenced by increases in the proportions of splenic T lymphocytes (CD3^+^ T cells) and T-cell subsets (CD8^+^ T cells), as well as the secretion of the T-cell-related cytokines interleukin (IL)-2, interferon-γ, IL-4 and granzyme B. These effects were associated with a reduction in oxidative stress, as evidenced by changes in the levels of hydroxyl radical, superoxide dismutase, glutathione peroxidase and malondialdehyde. Moreover, WPE decreased the FNT-induced overexpression of NADPH oxidase 2 and dual oxidase 1 by regulating Toll-like receptor 4 signaling in splenic T-cells. Taken together, these findings suggest that WPE protects against FNT-mediated immunotoxicity and improves immune function by inhibiting oxidative stress.

## 1. Introduction

Organophosphate pesticides (OPs) are extensively used in agriculture (most vegetables, fruits and forage crops) worldwide [1,2]. These pesticides and their metabolites are distributed widely in environment and food [3,4] and exhibit neurotoxicity, reproductive toxicity, respiratory toxicity, hepatotoxicity and immunotoxicity [3,5,6,7,8]. The OP fenitrothion (FNT)—*O*,*O*-dimethyl *O*-(3-methyl-4-nitrophenyl) phosphorothioate—is used to control insects on cereals, orchard fruits and vegetables and in forests [6,9]. However, FNT significantly reduces the spleen/body and thymus/body weight ratios [10], decreases splenic T-lymphocyte proliferation [11] and inhibits the production of T cell derived cytokines interleukin (IL)-2 and interferon-γ (IFN-γ) [12], indicating immunotoxicity. The toxicity of FNT is associated with the overproduction of reactive oxygen species (ROS) and the resulting oxidative stress [9,13,14].

Polyphenols confer protective effects against a variety of toxins. Quercetin, one of the most common polyphenols, significantly attenuated cadmium-induced cytotoxicity in rats [15], inhibited apoptosis and oxidative stress in chicken granulosa cells [16] and mitigated atrazine-induced oxidative stress in cultured rat interstitial Leydig cells [17]. Proanthocyanidin protected against nodularin-induced oxidative toxicity in *Carassius auratus* lymphocytes [18] and inhibited lead-induced neurological impairment [19]. Oleuropein alleviated oxidative stress and DNA damage caused by malathion in rats [20]. Polyphenols from the medicinal plant *Antirhea borbonica* exerted anti-inflammatory and antioxidant effects on lipopolysaccharide (LPS)-exposed adipocytes by reducing the Toll-like receptor (TLR)-dependent production of myeloid differentiation primary response 88 (MyD88) and nuclear factor kappa B (NF-κB) and NADPH oxidase 2 (NOX-2)–derived ROS [21]. However, the ability of these compounds to prevent FNT-induced immunotoxicity is unclear.

Walnuts are rich in polyphenols such as flavonoids and phenolic acid and are thus considered a “superfood” [22]. Walnut extracts exert antibacterial, anticancer, hepatoprotective, antidiabetic, anti-inflammatory, antidepressive and antioxidant effects [23]. Walnut polyphenols protected against 4-pentylphenol– and 3-methyl-4-nitrophenol–induced immunotoxicity, cigarette smoke extract-induced acute lung toxicity, cisplatin-induced disruptions in motor and cognitive functions in rats and carbon tetrachloride-mediated liver injury in mice [24,25,26,27]. However, the effects of walnut polyphenols on pesticide-induced immunotoxicity are unclear. Here, we investigated the protective effect of walnut polyphenol extract (WPE) on FNT-induced immunotoxicity and assessed the underlying mechanisms.

## 2. Materials and Methods

### 2.1. Materials

Walnuts were purchased from the Jingpin Fruit Industry Co., Ltd. (Hebei, China). FNT was obtained from AccuStandard (New Haven, CT, USA). RPMI 1640 medium was purchased from Mediatech (Manassas, VA, USA). Enzyme-linked immunosorbent assay (ELISA) kits (of mouse IL-2, IL-4, IFN-γ, IL-6 and granzyme B) were purchased by Huamei Biotech (Wuhan, China). The following antibodies purchased from Biogems (PeproTech, NJ, USA) were used in the phenotypic analysis studies: Fluorescein isothiocyanate (FITC)-labeled rat IgG2a and IgG2b (negative isotype controls) were obtained from Bio Legend (San Diego, CA, USA). FITC-labeled anti-mouse CD3^+^ (lgG2b), FITC-labeled anti-mouse CD8^+^ (lgG2b), FITC-labeled anti-mouse CD4^+^ (lgG2b) and FITC-labeled anti-mouse CD19^+^ (lgG2a). Assay kits of superoxide dismutase (SOD), glutathione peroxidase (GSH-Px), hydroxyl radical (•OH) and malondialdehyde (MDA) were obtained from Nanjing Jiancheng Bioengineering Institute (Nanjing, China). All other chemicals used here, such as NH_4_Cl, Concanavalin A (Con A) and LPS, etc. were purchased from Sigma (St. Louis, MO, USA). Rabbit anti-NOX-2 antibody, rabbit anti-DUOX-1 antibody, rabbit anti-TLR-4 antibody and secondary antibody (horseradish peroxidase [HRP]-linked anti-rabbit IgG) were obtained from Bioss Biotechnology Co. (Bejing, China).

### 2.2. Extraction of Polyphenols

The WPE was extracted by the protocols described in Yang et al. [24]. 30 g walnuts were frozen for over 24 h; the shelled kernels were ground and then immersed in acetate buffer (100 mM, pH 4.8)/acetone (30:70, *v*/*v*, 240 mL) for 24 h at 4 °C (the process was repeated). The extracts were concentrated at 37 °C under reduced pressure until the organic solvent was completely evaporated using a rotary evaporator. The concentrated solution was extracted three times with 75 mL ethyl acetate. These ethyl acetate extracts were evaporated to remove ethyl acetate, lyophilized, the powder was WPE. The total phenolic was measured using the Folin–Ciocalteu assay prescribed by Wang et al. [28]. The result was expressed as g of gallic acid equivalents (GAE)/g of sample.

### 2.3. Experimental Animals

8 weeks old specific-pathogen-free male Kunming mice (weight, 20.0 ± 2.0 g) were obtained from the Peking University Medical Department (Beijing, China). All mice were administrated in a pathogen-free facility with a 12-h light-dark cycle, which was maintained at a temperature of 23–25 °C and a relative humidity at 57–60%. They had *ad libitum* access to standard sterilized rodent chow and filtered water. All procedures here were reviewed and approved by the Policy on the Care and Use of Animals established by the Ethical Committee of the Beijing Forestry University that is fully accredited by the Department of Agriculture of Hebei Province, China (JNZF11/2007).

### 2.4. Preparation of Splenocytes

Splenocytes were prepared based on the protocols as previously described [24]. Five mice were euthanized by cervical dislocation, soaked with 75% alcohol for 3 min. Their spleens were removed and single cell suspensions were prepared by mincing and tapping spleen fragments on a stainless 200-mesh held in RPMI 1640, the medium was supplemented with 10% fetal bovine serum, 100 U penicillin/mL, 100 mg streptomycin/mL and 2 mM l-glutamine. Erythrocytes were lysed by incubating the cells in 0.8% *w*/*v* ammonium chloride solution on ice for 2 min. Followed centrifugation (380× *g*) for 5 min, the pelleted cells were washed three times and finally re-suspended in RPMI-1640. Cell viability was determined using trypan blue dye, which always exceeded 95%. In this study, splenocytes stimulated with Concanavalin A (Con A, 5 μg/mL) or LPS (10 μg/mL) were used in investigating proliferation of splenic T or B cell.

### 2.5. Cell Viability Assay

Cell viability was assayed as previously described [14]. 100 μL of splenocyte suspension (5 × 10^6^ cell/mL) was seeded into 96-well flat-bottom microtiter plates. After incubation 4 h, 100 μL FNT (10^−4^ M) alone or in combinations with WPE (0.5, 1.0, 5.0 and 10.0 µg/mL) was added to designated wells. Cells treated with complete medium were used as control. After another 48 h incubation at 37 °C in a humidified atmosphere with 5% CO_2_, 20 µL MTT (5 mg/mL) solution was added into each well. The samples were incubated a further 4 h. The culture supernatant was carefully removed and 200 µL DMSO was added to each well. The absorbance was measured at 570 nm using a microplate reader (BioRad, Hercules, CA, USA). Cell viability (% of control) = 100 × (the absorbance of experiment group/the absorbance of control group).

### 2.6. Flow Cytometry

Lymphocytes phenotypic analysis was performed by flow cytometry as described previously [11]. The cells were washed, then diluted to 2.5 × 10^7^ cells/mL in PBS. Cells were re-centrifuged and then re-suspended in 50 µL Ab Block at 4 °C for 5 min. The cells were blocked, then were added 1 µg/mL specific FITC-labeled antibody, then incubated at 4 °C in the dark for 30 min. After cell staining, the cells were washed with PBS three times and centrifugation. Then, the cells were transferred to FACS tubes (in PBS) for measures of CD3^+^, CD4^+^, CD8^+^ T-cells and B-cell subset levels by phenotypic analysis. The test was carried out by a BD FACS Calibur flow cytometer (Becton Dickinson, San Diego, CA, USA). Cells were excited with a 488 nm argon laser line and the fluorescence of FITC was analyzed on FL1 (530 nm), counting 10,000 events per sample. Splenocytes were electronically gated to exclude any residual platelets, red cells, or dead cell debris. The results were indicated as the percentage positive cells within a gate which was the same for both exposed and control splenocytes. All analyses were carried out with FlowJo software (Emerald Biotech, Hangzhou, China).

### 2.7. ELISA

Splenocytes were treated with the test reagents in 96-well plates (at 5 × 10^6^ cells/mL complete medium) for 48 h. Levels of IFN-γ, IL-2, IL-4, IL-6 and granzyme-B were measured by commercial ELISA kits. The levels of sensitivity of the kits were 3.9 pg IL-2/mL, 0.4 pg IL-4/mL, 0.39 pg IL-6/mL, 3.9 pg IFN-γ/mL and 3.1 pg granzyme-B/mL, respectively.

### 2.8. Measurement of GSH-Px, SOD, MDA and •OH Levels

Cells were treated with the test reagents in 96-well plates at a density of 5 × 10^6^ cells/mL for 48 h. Culture supernatants were collected and determined GSH-Px and SOD activities and MDA and •OH and MDA levels using commercial assay kits. The levels of sensitivity of the kits were 0.5 U GSH-Px/mL, 0.5 U SOD/mL, 0.01 mmoL MDA/mL and 0.04 U •OH/mL, respectively.

### 2.9. Western Blotting

Total cell extracts were prepared as previously described [29]. In brief, additional sets of cells were harvested and washed with PBS. Proteins were extracted with RIPA lysis buffer containing 10 mg/mL phenylmethanesulfonyl fluoride (PMSF) on ice for 30 min. After protein content determination, protein samples were resolved on SDS-PAGE (10–12% gels) and transferred to nitrocellulose membranes (Bio-Rad, Richmond, CA, USA). Membranes were blocked with 3% BSA for 1 h at room temperature and incubated overnight at 4 °C with primary antibodies against NOX-2, DUOX-1 and TLR-4. HRP-conjugated secondary incubation was then carried out. Finally, membranes were colored with 10 mg 3,3-diaminobenzidine (Wako, Tokyo, Japan) solution in 50 mL phosphate buffer (0.03 M) plus 3 μL H_2_O_2_. β-actin was used for the endogenous control. Bands were analyzed quantitatively using Quantity One software (Version 4.5, Bio-Rad Laboratories, Inc., Hercules, CA, USA).

### 2.10. Fractionation of WPE by Column Chromatography

In order to further study the active components in walnut polyphenols, 11 fractions (F1–F11) were separated from WPE by silica gel column chromatography using increasing polarity gradients of a mixture of methanol/chloroform (10:0, 9:1, 8:2, 7:3, 6:4, 5:5, 4:6, 3:7, 2:8, 1:9, 0:10 *v*/*v*). The eluent was evaporated in a rotary evaporator and powder of fractions was obtained by freeze-dried.

### 2.11. LC-MS Analyses (HPLC-ESI-IT-TOF-MS)

LC–MS analysis was carried out as previously described [24]. LC–MS analysis was conducted in a UPLC-Triple-TOF/MS system consisting of AcquityTM ultra high performance liquid chromatograph (HPLC) (Water, Milford, CT, USA), Triple TOF 5600+ Time of Flight Mass Spectrometer (TOF) and electrospray ion source (ESI). Chromatographic separation was performed on a ZORBAX-SBC18 chromatographic column (100 mm × 4.6 mm id × 1.8 µm, Agilent, CA, USA). Mobile phases were 0.1% formic acid in water (A) and 0.1% formic acid in acetonitrile (B). Before injection, the column was equilibrated for 5 min at initial conditions (5% B). The following condition was used: a step gradient of 5–95% B over 35 min. The injection volume and the flow rate were 5 µL and 0.8 mL/min. ESI source voltage was set at 4.5 kV in negative ionization mode maintained at 550 °C. Data was acquired from *m*/*z* 100–1500 and collected with TOF MS ~ Product Ion ~ IDA mode. Major phenolic compounds found in walnut extracts samples are shown in Table 1 and Table 2.

### 2.12. Statistical Analysis

At least three individual experiments were conducted for each experiment and satisfactory correlation was achieved between the results of each individual experiments. All data were expressed as means ± SD. Statistical analyses were performed using one-way analysis of variance (ANOVA) followed by a post hoc test, Tukey’s test with Graph Pad Prism software (Version 6.01, GraphPad, San Diego, CA). A *p*-value < 0.05 was accepted as significant, *p*-value < 0.01 was taken as very significant.

## 3. Results

### 3.1. WPE Protects Splenocytes against FNT Induced Cytotoxicity

**Figure 1:**
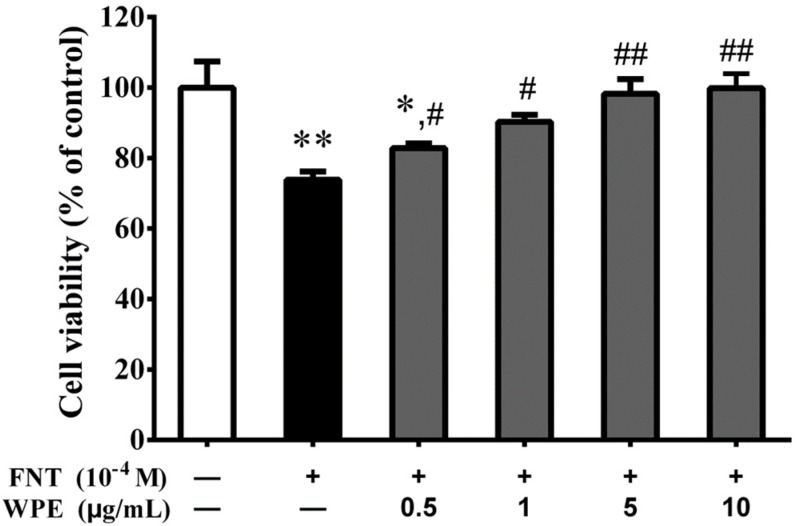
Effect of WPE on cytotoxicity in splenocytes exposed to FNT by MTT assay. Splenocytes were treated with fenitrothion (FNT, 10^−4^ M) or different concentrations (0.5, 1.0, 5.0 and 10.0 μg/mL) of WPE together with FNT. Results are presented as mean ± SD of three separate experiments. * *p* < 0.05 or ** *p* < 0.01, vs. untreated control; ^#^ *p* < 0.05 or ^##^ *p* < 0.01 vs. FNT treatment.

To investigate the effect of WPE on FNT exposed splenocytes, cell viability was measured using the MTT assay. As shown in Figure 1, FNT significantly decreased splenocyte viability to 73.8% of the control level (*p* < 0.01). WPE at 0.5–1.0 µg/mL inhibited cytotoxicity in a concentration dependent manner. Treatment of FNT exposed splenocytes with 1.0 µg/mL WPE increased their viability from 73.8% to 90.3% relative to the control. Because 1.0 µg/mL WPE resulted in significantly protective activity, the concentration of WPE was chosen for used in all subsequent experiments.

### 3.2. Effect of WPE on cytotoxicity of FNT in Splenic Lymphocyte Subpopulations

**Figure 2:**
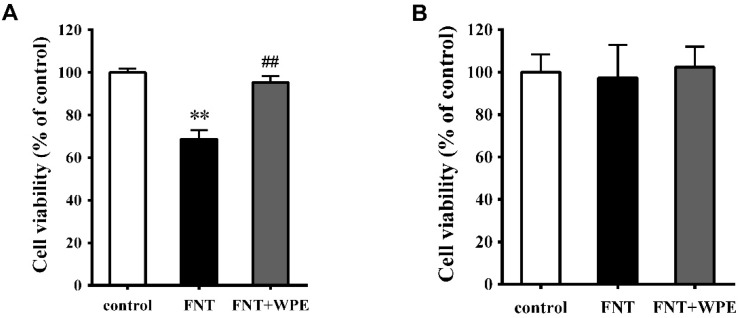
Effect of WPE on cytotoxicity of fenitrothion (FNT) in splenic lymphocyte subpopulations. Splenocytes were treated with FNT or WPE together with FNT for 48 h in the presence of Con A (5 μg/mL) or LPS (10 μg/mL). (**A**) Splenic cells stimulated with Con A (T lymphocytes) or (**B**) Splenic cells stimulated with LPS (B lymphocytes) viability was evaluated by an MTT assay. Controls were splenic cells cultured in medium contained Con A or LPS, untreated with FNT or WPE. Results shown are as mean ± SD of three separate experiments. ** *p* < 0.01, vs. control; ^##^ *p* < 0.01 vs. FNT treatment.

**Figure 3:**
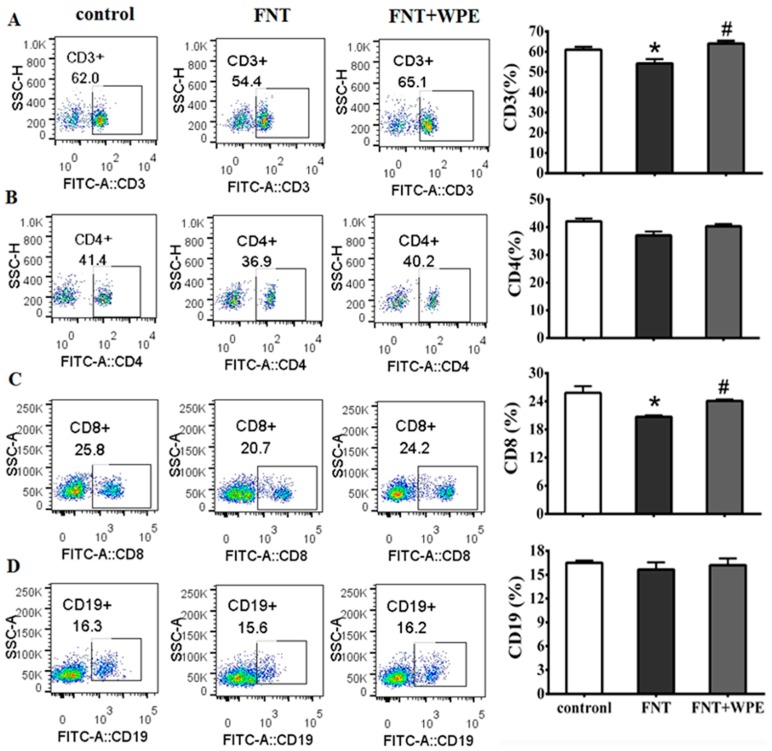
Percentages of various lymphocyte cell types as determined using flow cytometric analysis. (**A**) Cluster of differentiation (CD)3^+^ T-cells, (**B**) CD4^+^ T-cells, (**C**) CD8^+^ T-cells and (**D**) CD19^+^ B-cells in cells exposed to medium only (control), FNT or WPE together with FNT. Results shown are means ± SD of three separate experiments. * *p* < 0.05 vs. untreated control; ^#^ *p* < 0.05 vs. FNT treatment. (SSC: side scatter. FITC: fluorescein isothiocyanate.)

Treatment with appropriate concentration of Con A or LPS induced T-cells or B-cells proliferation, respectively, during splenic cells culturing [32,33]. In this study, splenocytes stimulated with Con A (5 μg/mL) or LPS (10 μg/mL) were used in investigating proliferation or function of splenic T or B cell. To investigate the effects of WPE on splenic T and B cell populations exposed to FNT, splenocytes were treated with FNT alone or in combination with WPE for 48 h in the presence of a mitogen (Con A or LPS) and cell viability was evaluated by MTT assay. As shown in Figure 2, FNT significantly inhibited the proliferation of splenic cells stimulated with Con A (T cells) but not that of splenic cells stimulated with LPS (B cells). However, the effect of FNT on splenic cells stimulated with Con A (T cells) was significantly attenuated by WPE treatment, as their viability increased from 69.3 to 94.6% (Figure 2A). To further investigate the effects of WPE on T cell populations exposed to FNT, the splenocytes were stained with FITC labeled antibodies (48 h) and assessed using flow cytometry. As shown in Figure 3A,C, the percentages of CD8^+^ and CD3^+^ T cells were significantly lower in splenocytes exposed to FNT relative to the control. However, FNT did not have a significant effect on CD4^+^ T-cells (*p* > 0.05) and decreased the cell populations (Figure 3B). FNT also did not affect the number of CD19^+^ B cells (Figure 3D). WPE increased the proportions of CD3^+^, CD4^+^ and CD8^+^ T-cells among FNT exposed splenocytes, with overall T cell numbers similar to the controls. These results suggest that WPE is capable of normalizing the proportions of T cell subpopulations among FNT exposed splenocytes.

### 3.3. WPE Protected Against Decreases in Splenic T Cell Cytokine/Granzyme Production

**Figure 4:**
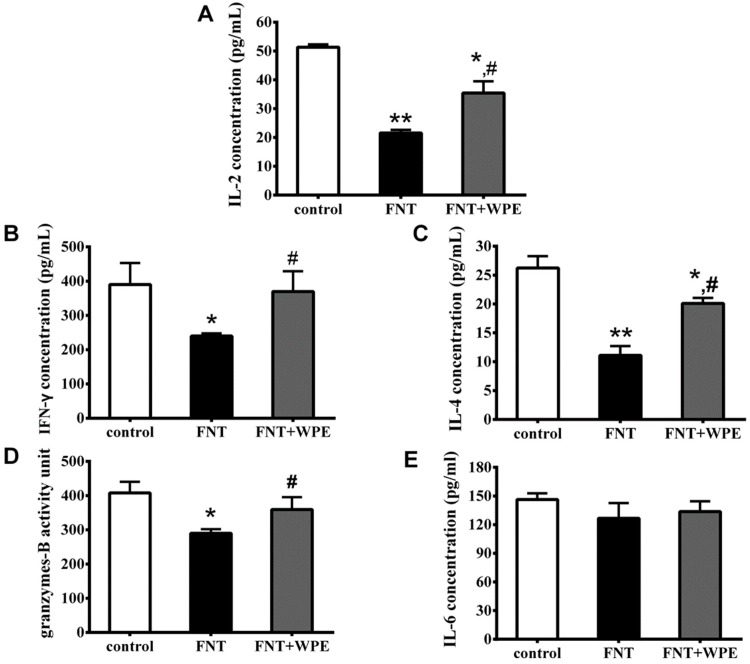
Effects of WPE on select cytokine/granzyme production in splenocytes exposed to FNT. Levels of (**A**) interleukin (IL)-2, (**B**) interferon (IFN)-γ, (**C**) IL-4, (**D**) granzyme B and (**E**)IL-6 released into culture media were then measured by ELISA. Results shown are means ± SD of three separate experiments. * *p* < 0.05 or ** *p* < 0.01 vs. untreated control; ^#^ *p* < 0.05 vs. FNT treatment.

To evaluate the effect of WPE on immune function of splenic lymphocyte, splenocytes were treated with FNT or WPE together with FNT for 48 h and cytokine and granzyme B production was determined by ELISA. T-cells mature into CD4^+^ T-cells (T helper [TH] cells) or CD8^+^ T cells; CD4^+^ TH cells consist of CD4^+^ TH1 and CD4^+^ TH2 phenotypes. IL-2 and IFN-γ are secreted by TH1 cells and IL-4 by TH2 cells. Granzyme B is produced by CD8^+^ T-cells and IL-6 by splenic B cells. As shown in Figure 4A–D, FNT significantly inhibited IFN-γ, IL-2, IL-4 and Granzyme B production from splenic T cells. Treatment with WPE significantly increased the secretion of IL-2, IFN-γ, IL-4 and granzyme B by FNT exposed cells from 41.7% to 68.9% (21.6 to 35.5 pg/mL), 61.4% to 94.8% (239.9 to 369.8 pg/mL), 42.3% to 76.9% (11.1 to 20.1 pg/mL) and 71.0% to 88.2% (289.7 to 359.4 units), respectively, relative to controls. Therefore, WPE protects splenocytes from the FNT induced decrease in T cell cytokine/granzyme production.

### 3.4. WPE Attenuates FNT Induced Oxidative Damage in Splenocytes

**Figure 5:**
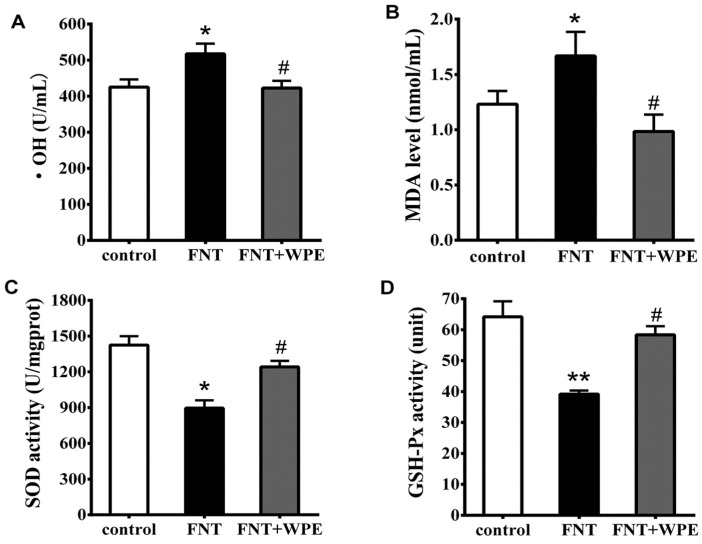
Effects of WPE on the oxidative stress parameters in splenic cells exposed to FNT. Changes in (**A**) hydroxyl radical (•OH) content, (**B**) malondialdehyde (MDA) content, (**C**) superoxide dismutase (SOD) activity and (**D**) glutathione peroxidase (GSH-Px) activity in the cells were measured using specific assay kits. Controls were splenic cells cultured in medium, untreated with FNT or WPE. Results shown are means ± SD of three separate experiments. * *p* < 0.05 or ** *p* < 0.01 vs. untreated control; ^#^ *p* < 0.05 vs. FNT treatment.

To explore whether WPE inhibits FNT induced oxidative stress, the levels of •OH, MDA, SOD and GSH-Px in FNT exposed splenocytes were evaluated. As shown in Figure 5, FNT significantly increased the levels of •OH and MDA and decreased the activities of SOD and GSH-Px relative to the controls. However, treatment with WPE reduced the levels of •OH and MDA from 121.6% to 99.2% (296.6 to 261.2 U/mL) and from 136.0% to 80.3% (2.50 to 0.98 nmol/mL), respectively, relative to controls (Figure 5A,B). In contrast, treatment with WPE increased the activities of SOD and GSH-Px from 62.9% to 87.1% (896.1 to 1241.8 U/mg prot) and from 60.5% to 89.9% (39.2 to 58.4 units), respectively, relative to controls (Figure 5C,D).

**Figure 6:**
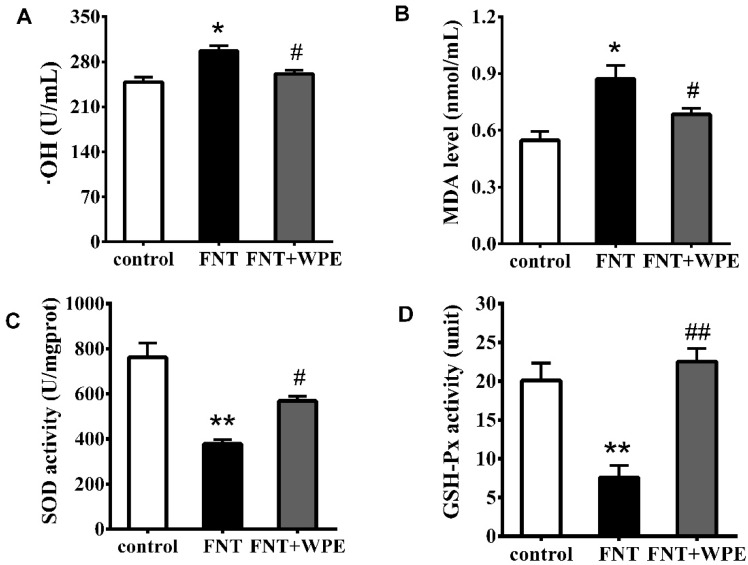
Effects of WPE on changes of the oxidative stress parameters induced by FNT in splenic cells stimulated by with Con A (5 μg/mL). Changes in (**A**) •OH content, (**B**) malondialdehyde (MDA) content, (**C**) SOD activity and (**D**) GSH-Px activity in the cells were measured using specific assay kits. Controls were splenic cells cultured in medium contained Con A or LPS, untreated with FNT or WPE. Results shown are means ± SD of three separate experiments. * *p* < 0.05 or ** *p* < 0.01 vs. control; ^#^ *p* < 0.05 or ^##^ *p* < 0.01 vs. FNT treatment.

FNT induced significant oxidative damage in splenic cells stimulated by with Con A [14]. To investigate whether WPE attenuates FNT induced oxidative stress in the splenic cells, splenocytes were treated with FNT alone or in combination with WPE for 48 h in the presence of Con A. As shown in Figure 6, the levels of •OH and MDA were increased and the activities of SOD and GSH Px were significantly inhibited in splenic T-cells stimulated with Con A. However, treatment with WPE significantly reduced oxidative stress in the splenic T lymphocytes, as evidenced by an increase in the levels of •OH and MDA from 119.5% to 105.0% (296.6 to 261.2 U/mL) and from 159.3% to 125.0% (0.87 to 0.69 nmol/mL), respectively, relative to controls (Figure 6A,B). In contrast, treatment with WPE increased the activities of SOD and GSH-Px from 49.7% to 74.5% (379.3 to 568.6 U/mg prot) and from 37.9% to 104.8% (7.60 to 22.53 units), respectively, relative to controls. These results suggest that WPE prevents FNT induced oxidative damage in Con A induced splenic T-cells.

### 3.5. WPE Inhibits the Expression of NOX-2, DUOX-1 and TLR-4 in Splenic Cells

**Figure 7:**
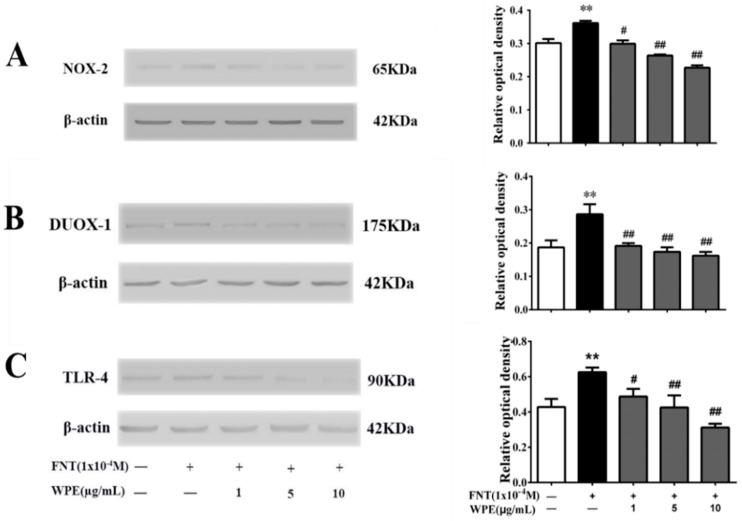
Effects of WPE on the expression of nicotinamide adenine dinucleotide phosphate (NADPH) oxidase and TLR-4 in Splenic T Cells exposed to FNT in the presence of Con A (5 μg/mL). The expression of (**A**) NOX-2, (**B**) DUOX-1 and (**C**) TLR-4 in splenic T cells were measured using western blotting. Results shown are means ± SD of three separate experiments. ** *p* < 0.01 vs. untreated control; ^#^ *p* < 0.05 or ^##^ *p* < 0.01 vs. FNT treatment.

Some dietary polyphenols can alleviate ROS production by suppressing the activation of intracellular nicotinamide adenine dinucleotide phosphate (NADPH) oxidase (NOX) [34,35]. The NOX family comprises seven members: NOX-1, NOX-2, NOX-3, NOX-4, NOX-5, dual oxidase (DUOX)-1 and DUOX-2 [36,37]. NOX-2 and DUOX-1 are expressed on T-cells [38,39]. To assess the mechanism underlying the protective effect of WPE on FNT induced immunotoxicity, we evaluated the expression of NOX-2 and DUOX-1 on splenocytes treated with FNT alone or in combination with WPE for 48 h in the presence of Con A. As shown in Figure 7A,B, WPE at 1–10 μg/mL significantly reduced NOX-2 and DUOX-1 overexpression induced by FNT in Con A stimulated splenic T-cells in a concentration dependent manner.

TLRs play a crucial role in recognizing environmental stresses and pathogen-associated molecular patterns [40]. TLR-4 is related to the activation of NOX and has been reported to express in T cells [41,42] and expression of TLR-4 in immune cells activates NOX-2, leading to ROS generation [43]. As shown in Figure 7C, FNT increased the expression of TLR-4, which was significantly reduced by ≥1 µg/mL WPE in a dose dependent manner. Thus, WPE inhibits overactivation of NOX-2 and DUOX-1 by suppressing the overexpression of TLR-4 induced by FNT in Con A stimulated splenic T-cells.

### 3.6. Identification of Phenolic Compounds in WPE

**Figure 8:**
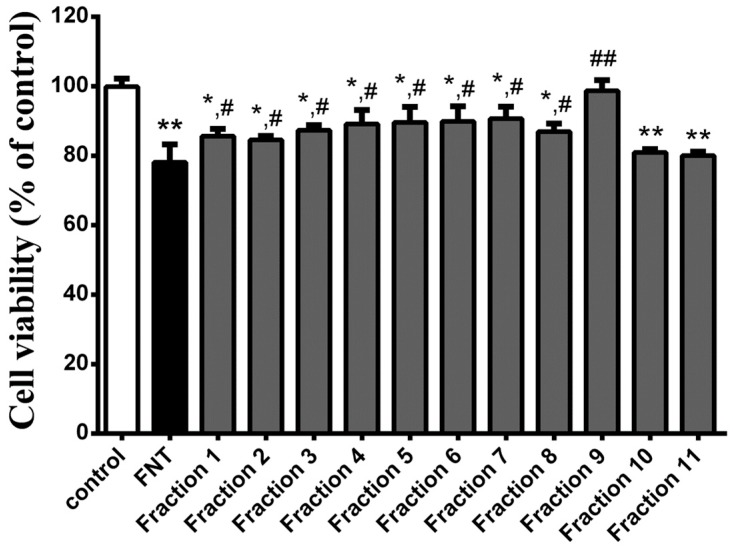
Effects of the 11 fractions on FNT induced immunotoxicity in splenocytes using MTT assay. Results shown are means ± SD of three separate experiments. * *p* < 0.05 or ** *p* < 0.01 vs. untreated control; ^#^ *p* < 0.05 or ^##^ *p* < 0.01 vs. FNT treatment.

WPE exerts a strong antioxidant effect. A Folin–Ciocalteu phenol assay showed that the average total polyphenol content of the extracts was 76.30 ± 1.21 mg GAE/g. Next, we examined the phenolic constituents of WPE by liquid chromatography–mass spectrometry (LC–MS); the names and *m*/*z* scores of the compounds are listed in Table 1. A total of 19 phenolic compounds was tentatively identified, including ellagitannins (1–5, 7–10, 12, 14, 15, 17), gallic tannins (11, 13, 18), phenolic acid (16) and flavonoids (6, 19). To identify the active components, WPE was fractionated into 11 fractions by silica gel column chromatography with an increasing polarity gradient of a methanol/chloroform mixture. The protective effects of the 11 fractions against FNT induced immunotoxicity in splenocytes were evaluated by MTT assay. Fraction 9 exhibited the greatest immunoprotective effect, increased cell viability from 77.7% to 98.3% in FNT exposed splenocytes, relative to control (Figure 8). LC–MS showed that fraction 9 contained the polyphenolic compounds ellagic acid, ellagic acid pentose isomer, ellagic acid hexose isomer and quercetin (Table 2).

## 4. Discussion

We investigated the effect of WPE on FNT induced cytotoxicity in mouse splenocytes in vitro. WPE attenuated FNT mediated toxicity, as evidenced by significant increases in cell viability and the proportions of CD3^+^, CD4^+^ and CD8^+^ T cells. These WPE induced increases in T cell populations were accompanied by marked increases in the production of IL-2, IFN-γ, IL-4 and Granzyme B. Evaluation of the levels of •OH, MDA, SOD and GSH-Px showed that WPE significantly decreased FNT induced oxidative damage in splenic cells by suppressing the overexpression of NOX-2, DUOX-1 and TLR-4. LC-MS showed that WPE comprised at least 19 phenolic compounds and the WPE fraction with the greatest activity contained ellagic acid, ellagic acid pentose isomer, ellagic acid hexose isomer and quercetin.

The ability of natural products to prevent the unfavorable health effects of xenobiotics is of increasing interest [44]. Polyphenols are reported to protect against the toxicity of pesticides and their metabolites. A polyphenol compound from honey alleviated the mitochondrial dysfunction and DNA damage induced by glyphosate and the organophosphate insecticide chlorpyrifos [45]. Quercetin significantly attenuated atrazine induced toxicity to interstitial Leydig cells in rats by restoring the expression of NF-κB and steroidogenic activity, as well as reducing oxidative stress [17]. Walnut polyphenols attenuated the 3-methyl-4 nitrophenol (the major metabolite of FNT) induced reproductive toxicity in chicken spermatogonial cells [24,46,47]. In this study, treatment with WPE significantly attenuated the FNT induced cytotoxicity in mouse splenocytes in vitro.

T-cells comprise a large proportion of splenic lymphocytes and are critical for cell mediated immunity. Polyphenols can maintain the functionality of T-cells. Treatment of *Pennisetum glaucum* seeds with polyphenols modulated the growth of rat T lymphocytes [48]. Similarly, polyphenols in chocolate regulated the proliferation of T-cells from human peripheral blood, whereas walnut polyphenols normalized the proportions of T cell subpopulations among murine splenic lymphocytes exposed to 4-pentylphenol and 3-methyl-4-nitrophenol [49]. In this study, WPE restored the proportions of T cell subpopulations to the values of the controls. These results are in general agreement with previous reports that phenolic compounds can maintain the functionality of T-cells.

Cytokines activate and modulate the function of immune cells [50] and dietary polyphenols influence cytokine secretion by immune/non-immune cells [51,52,53,54]. Curcumin, a natural polyphenol from the root of *Curcuma longa*, by modulating the expression of IFN-α, TNF-γ, IL-17 and IL-10, regulated the proportions of the various TH subsets, which ameliorated experimental autoimmune myasthenia gravis in rats [51]. Similarly, in vivo curcumin decreased the levels of IFN-γ and IL-1β in TH1 cells, which may protect against nicotine induced toxicity during protein malnutrition in rats [52]. Similarly, lychee fruit polyphenols exerted an anti-inflammatory effect by decreasing the secretion of proinflammatory cytokines (including IL-6 and TNF-α) by monocytes from peripheral blood [53]. Moreover, polyphenols from red grapes protected against loss of immune function in sea bass exposed to microbes and environmental stresses by increasing the levels of IFN-γ [54]. In this study, WPE increased the production of T cell–related cytokines by FNT exposed splenic lymphocytes, consistent with the above mentioned previous reports.

We identified 19 phenolic compounds, including ellagitannins, gallic tannins, phenolic acid and flavonoids, in WPE. The WPE fraction containing ellagic acid, ellagitannins (ellagic acid pentose isomer and ellagic acid hexose isomer) and quercetin exerted the greatest protective effect. Ellagic acid inhibited paraquat induced oxidative stress in A549 human alveolar cells [55]. Ellagitannins from *Phyllanthus amarus* attenuated liver injury in mice by reducing ethanol induced cytotoxicity and apoptosis [56] and quercetin protected against ATZ induced cytotoxicity by reducing oxidative stress in interstitial Leydig cells from immature rats [17]. In this study, ellagic acid, ellagitannins (ellagic acid pentose isomer and ellagic acid hexose isomer) and quercetin ameliorated FNT induced immunotoxicity in murine splenic lymphocytes.

Exposure to hazardous substances, including organic pollutants, can result in ROS overproduction, leading to oxidative stress [57]. Increased oxidative stress results in lipid peroxidation, protein oxidation and DNA damage [58]. However, dietary polyphenols can protect against oxidative stress. Curcumin, a natural polyphenol, decreased oxidative damage in zymosan induced multiple organ dysfunction syndrome by increasing the activity of SOD and reducing the concentration of MDA [59], whereas supplementation with walnut polyphenols exerted similar effects in the brain tissues of mice with hypercholesterolemia [60]. Similarly, pomegranate polyphenols, which have remarkable antioxidant properties, reduced ROS generation and ameliorated arsenic induced hepatic damage [61]. In this study, WPE significantly decreased oxidative damage in FNT exposed splenic T cells by increasing the activity of SOD and GSH-Px and decreasing the levels of •OH and MDA, which improved the functionality of the cells.

NADPH oxidase catalyzes the biosynthesis of ROS (O_2_^•−^ and H_2_O_2_) [62,63]. Environmental toxicants induce the phosphorylation of NADPH oxidase and its membrane translocation, resulting in oxidation of NADPH and transfer of its electrons to oxygen, forming ROS (O_2_^•−^) [36,64]. Organochlorine insecticides (trans nonachlor, dieldrin and dichlorodiphenyldichloroethylene) also activate NADPH oxidase and oxidative stress in human monocytes [37]. Polyphenols exert an antioxidant effect by modulating the expression and activity of NADPH oxidase [34,35]. Polyphenols reportedly decrease the production of ROS by inhibiting the expression, membrane translocation, activation and assembly of the NADPH oxidase complex [34,35,37]. NADPH oxidase is a multi-subunit holoenzyme that consists of two membrane-bound components, gp91phox and p22phox and the cytosolic regulatory subunits Rac, p67phox, p40phox and p47phox [36]. In NADPH oxidase, gp91phox is the major catalytic subunit and its homologs NOX-1, NOX-2, NOX-3, NOX-4, NOX-5, DUOX-1 and DUOX-2 [36,37] are expressed in different cell types [65]. Polyphenols exert an antioxidant effect by inhibiting the expression of different homologs of gp91phox [34,35,64]. Polyphenols from olive oil decreased vascular endothelial growth factor mediated ROS generation by suppressing the expression of NOX-2 and NOX-4 [34]. Polyphenols from French red wine restored the expression of NOX-1 to a level similar to that in young rats [64]. Pterostilbene, an analog of resveratrol, significantly alleviated subarachnoid hemorrhage induced oxidative stress by modulating the expression of NOX-2 in C57BL/6 J mice [35]. T cells express NOX-2 and DUOX-1, which catalyze the reduction of molecular oxygen to generate superoxide O_2_^•−^, which can dismute to generate ROS species in T lymphocytes [38,39]. In the both NADPH oxidase, NOX-2 involve in ROS generation in T cells of human or animal under inflammatory conditions [42]. In this study, FNT induced oxidative stress by enhancing expression of NOX-2 and DUOX-1 in splenic cells stimulated with Con A (Figure 7A,B), resulted in the immunotoxicity, such as inhibition of proliferation and production of T cell–related cytokines (Figure 1, Figure 2, Figure 3 and Figure 4). However, WPE significantly protected against FNT induced oxidative damage by suppressing the expression of NOX-2 and DUOX-1 in splenic cells stimulated with Con A, in agreement with the above mentioned previous reports.

TLR-4 is a pathogen associated molecular pattern receptor and plays a critical role in the induction of the innate and adaptive immune responses [66]. TLR-4 not only was expressed in phagocytic cell but also was expressed in T cells of human and mice [41,42]. TLR-4 is related to the activation of NADPH oxidase, TLR-4 activation seemed to be a prerequisite for NAPDH oxidase activation, which regulated NOX-2 derived ROS generation via NF-κB pathway [42,66,67]. TLR-4 deficient animals decreased p47phox phosphorylation and reduced NADPH oxidase activation [66]. Indeed, polyphenols modulate NADPH oxidase expression by regulating TLR-4 signaling [21,67]. Polyphenols from the medicinal plant *Antirhea borbonica* decreased NOX-2–mediated ROS generation in LPS exposed adipocytes by reducing the TLR dependent production of MyD88 and NF-κB [21]. Similarly, curcumin suppressed LPS induced NOX mediated ROS production by inhibiting TLR-4 signaling in rat vascular smooth muscle cells [67]. In this study, WPE decreased the FNT induced overexpression of TLR-4, suggesting that its normalization of the levels of NOX-2 and DUOX-1 was mediated by the regulation of TLR-4 signaling in splenic T-cells.

## 5. Conclusions

We report here that WPE protected against FNT mediated toxicity in splenic T-cells, as evidenced by increases in cell viability and cytokine/granzyme production in vitro. The protective effect of WPE in FNT exposed splenic T-cells was due at least in part to attenuation of oxidative damage mediated by suppression of the activation of NOX-2 and DUOX-1, the latter caused by a reduction in the expression of TLR-4. Further studies of the mechanisms underlying these protective effects of walnut polyphenols are warranted.

## Figures and Tables

**Table 1:** Identification of phenolic compounds in walnut polyphenol extract (WPE) using HPLC-ESI-IT-TOF-MS ^1^ in the negative ion mode.

No.	t_R_ (min)	Measured [M-H]^−^ (*m*/*z*)	Predicted [M-H]^−^ (*m*/*z*)	MS/MS ^2^ Fragments (*m*/*z*)	Identification	Molecular Formula	Reference
1	5.02	783.0773	783.0681	481.06, 300.99, 275.02	Pedunculagin/casuariin isomer (bis-HHDP-glucose)	C_34_H_24_O_22_	[30,31]
2	7.14	951.0762	951.0740	907.08, 783.07, 481.06, 300.99, 275.02	PraecoxinA/ platycariin isomer (trigalloyl-HHDP-glucose)	C_41_H_28_O_27_	[30]
3	8.31,10.14, 11.12, 11.89, 12.75	785.0845	785.0840	633.07, 483.08, 300.99, 275.02	Tellimagrandin I isomer (digalloyl-HHDP-glucose)	C_34_H_26_O_22_	[30,31]
4	8.96	633.0763	633.0720	463.05, 300.99 275.02	Strictinin/isostrictinin isomers (galloyl-HHDP-glucose)	C_27_H_22_O_18_	[30,31]
5	9.45, 12.29, 14.16	935.0813	935.0786	783.07, 633.07, 300.99, 275.02	Tellimagrandin I isomer (digalloyl-HHDP-glucose)	C_34_H_26_O_22_	[30]
6	9.79	301.0417	301.0347	151.04	Quercetin	C_15_H_10_O_7_	[30,31]
7	10.52, 11.60, 12.96, 16.24	933.0655	933.0630	631.06, 481.06, 450.99, 300.99	Glansrin C isomer	C_41_H_26_O_26_	[30,31]
8	10.67	907.0862	907.0837	783.07, 764.05, 481.06, 300.99, 275.02	Heterophylliin E isomer	C_40_H_28_O_25_	[30]
9	10.86	469.0040	469.0049	425.01, 300.99, 166.99	Valoneic acid dilactone/Flavogallonic acid dilactone isomer	C_21_H_10_O_13_	[30]
10	11.27	463.0512	463.0517	300.99	Ellagic acid hexoside isomer	C_20_H_16_O_13_	[30,31]
11	11.47, 12.13	635.0882	635.0877	483.08, 465.07, 423.06, 313.06, 169.01	Trigalloyl-glucose isomer	C_27_H_24_O_18_	[30]
12	12.55	1103.0873	1103.0850	1059.09, 935.07, 757.09, 633.07, 300.99	Rugosin C/platycaryanin A/glansrin A isomer	C_48_H_32_O_31_	[30]
13	13.14, 14.02, 14.32	787.0996	787.0996	635.08, 465.07, 169.01	Tetragalloyl-glucose	C_34_H_28_O_22_	[30]
14	13.39, 15.11	937.0956	937.0947	785.08, 635.08, 483.07, 300.99	Tellimagrandin II/pterocaryanin C isomer	C_41_H_30_O_26_	[30]
15	13.42	433.0399	433.0405	300.99	Ellagic acid pentoside isomer	C_19_H_14_O_12_	[30,31]
16	14.49	300.9989	300.9989	283.99, 257.01, 229.01, 185.02	Ellagic acid	C_14_H_6_O_8_	[30,31]
17	15.49, 18.90	1085.0771	1085.0740	783.07, 633.07, 450.99, 300.99	Eucalbanin A/cornusiin B isomer	C_48_H_30_O_30_	[30]
18	15.72	939.1120	939.1100	787.09, 769.09, 617.08	1,2,3,4,6-Pentagalloyl-glucose	C_41_H_32_O_26_	[30]
19	17.03	433.1127	433.0772	300.03, 301.33	Quercetin pentoside isomer	C_20_H_18_O_11_	[30,31]

^1^ HPLC-ESI-IT-TOF-MS: High performance liquid chromatograph-electrospray ion source-ion trap-time of flight mass spectrometer- mass spectrometry. ^2^ MS/MS: Tandem mass spectrometry.

**Table 2:** Identification of fraction 9 using HPLC-ESI-IT-TOF-MS in the negative ion mode.

No.	t_R_ (min)	Measured [M-H]^−^ (*m*/*z*)	Predicted [M-H]^−^ (*m*/*z*)	MS/MS Fragments (*m*/*z*)	Identification	Molecular Formula	Reference
1	9.79	301.0417	301.0347	150.00	Quercetin	C_15_H_10_O_7_	[30,31]
2	11.27	463.0512	463.0517	300.99	Ellagic acid hexoside isomer	C_20_H_16_O_13_	[30,31]
3	13.42	433.0396	433.0405	300.99	Ellagic acid pentoside isomer	C_19_H_14_O_12_	[30,31]
4	14.49	300.9990	300.9990	283.99, 257.01, 229.01	Ellagic acid	C_14_H_6_O_8_	[30,31]
